# Supplementary figures and images for: Repair of mismatched templates during Rad51-dependent Break-Induced Replication
Source: PLoS Genet. 2022 Sep 2;18(9):e1010056. doi: 10.1371/journal.pgen.1010056 (PMC9477423; doi:10.1371/journal.pgen.1010056)

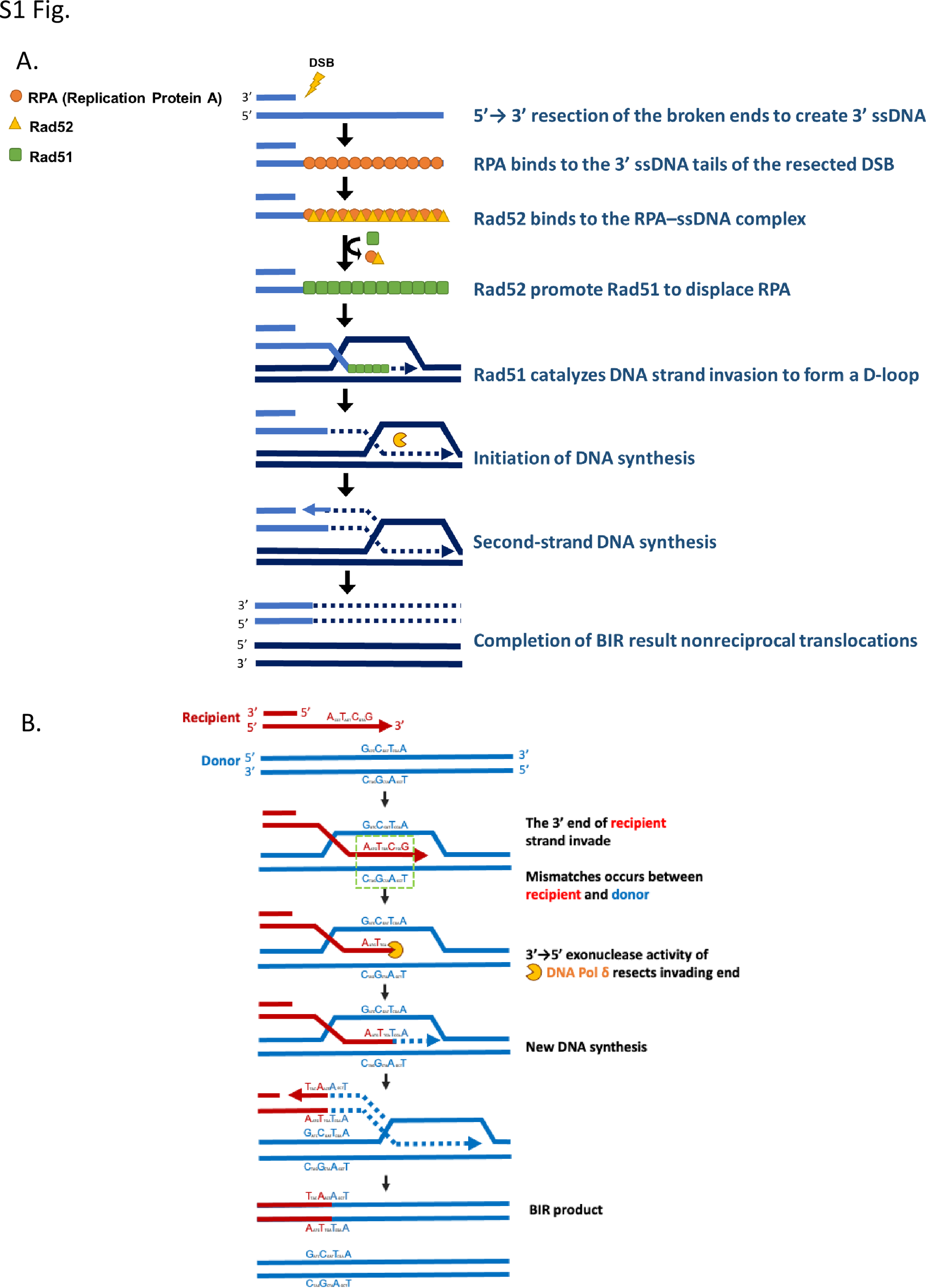

Supplement: S1 Fig — A. Mechanism of Rad51-dependent BIR B. Mismatch incorporation of heteroduplex DNA formation during BIR. Once a DSB is created, a broken end of DSB will be resected by 5’→3’ exonuclease to generate 3’ single-stranded DNA (ssDNA) which interacts with Rad51 and other recombination proteins to carry out homology search and strand invasion. The resected end of recipient sequence (indicated in red) will synapse with the donor (indicated in blue). Mismatches in the heteroduplex region during strand invasion are apparently not corrected by the Msh2/Mlh1 mismatch repair complex; rather DNA polymerase δ is recruited to the 3’ end and performs its proofreading 3’→5’ exonuclease activity prior to initiating new DNA synthesis from the 3’ invading end. DNA Polymerase δ “can apparently “back up” into the heteroduplex region as far as 40–50 nt and resynthesizes the region, copying the donor template sequences into the recovered BIR product. (TIFF) [file pgen.1010056.s001.tiff]

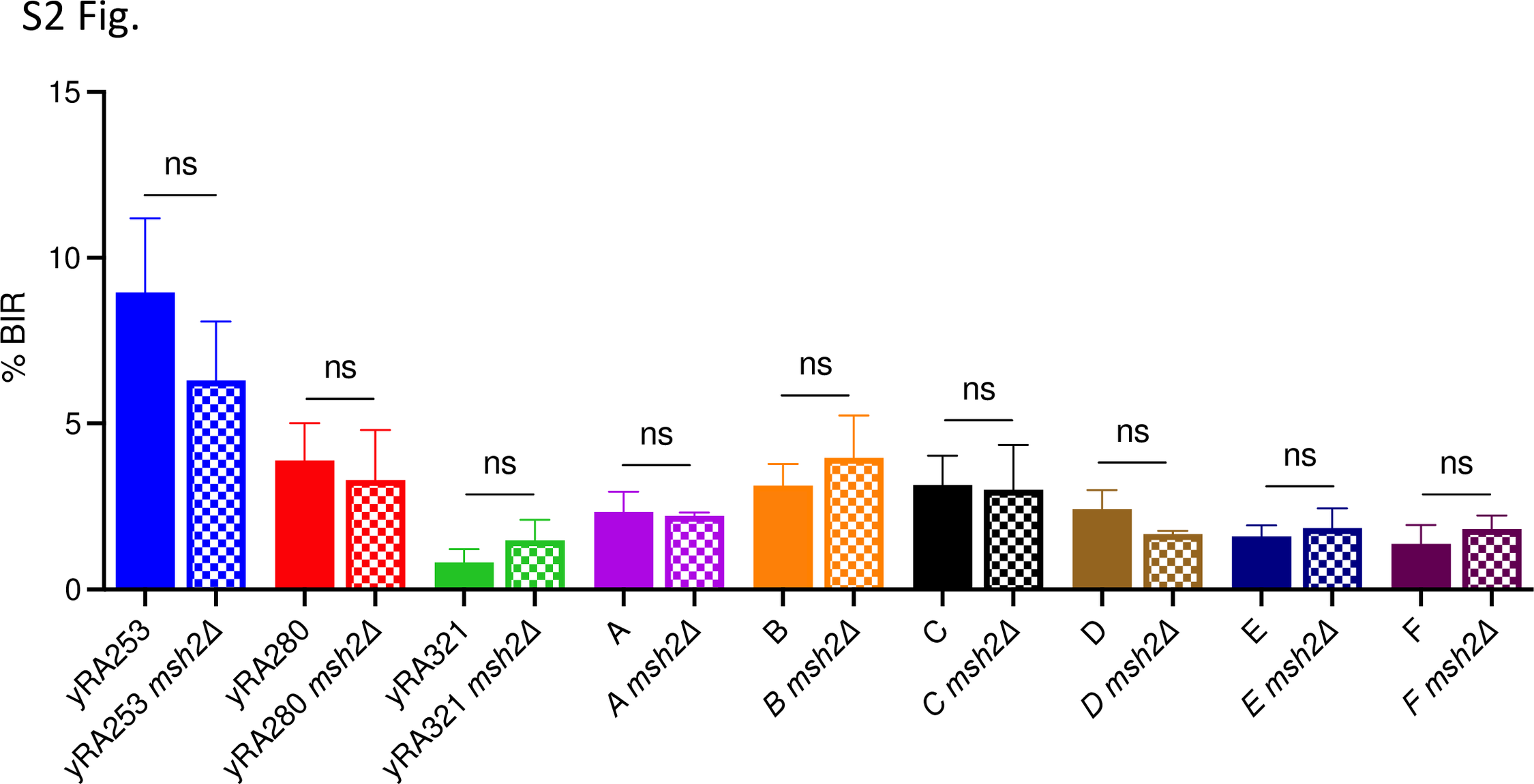

Supplement: S2 Fig — Wild type and msh2Δ derivatives of each donor template were measured as described in Fig 1. Statistical significance of the differences for each donor/msh2Δ pair was determined using an unpaired t-test with Welch’s correction. Error bars refer to standard deviation. Each measurement is based on a minimum of three experiments. (TIFF) [file pgen.1010056.s002.tiff]

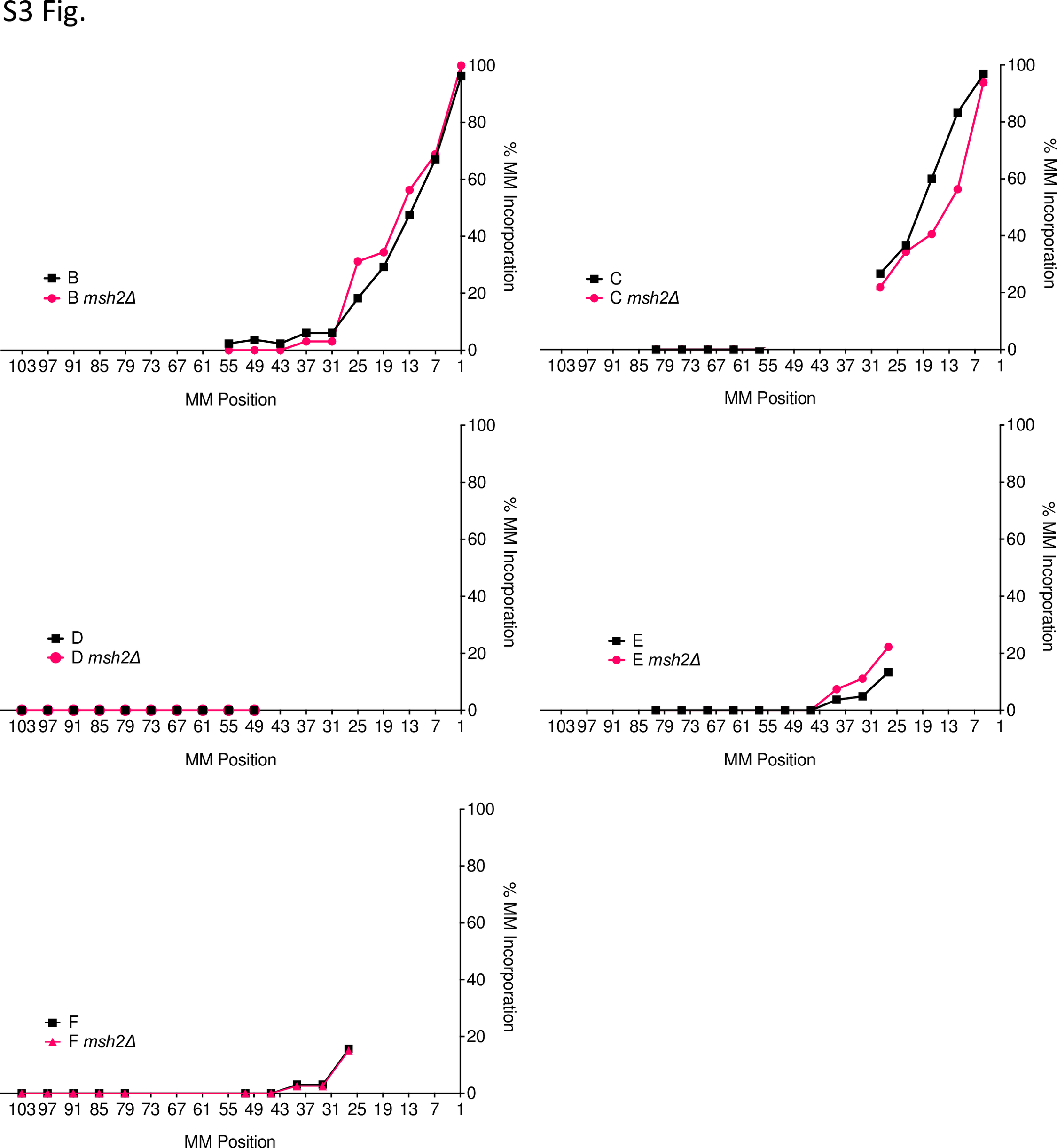

Supplement: S3 Fig — The effect of deleting mismatch repair gene MSH2 on mismatch incorporation pattern and BIR efficiency for donor templates with uneven mismatch distribution. For all % mismatch incorporation data, a minimum of 40 samples were DNA sequenced. (TIFF) [file pgen.1010056.s003.tiff]

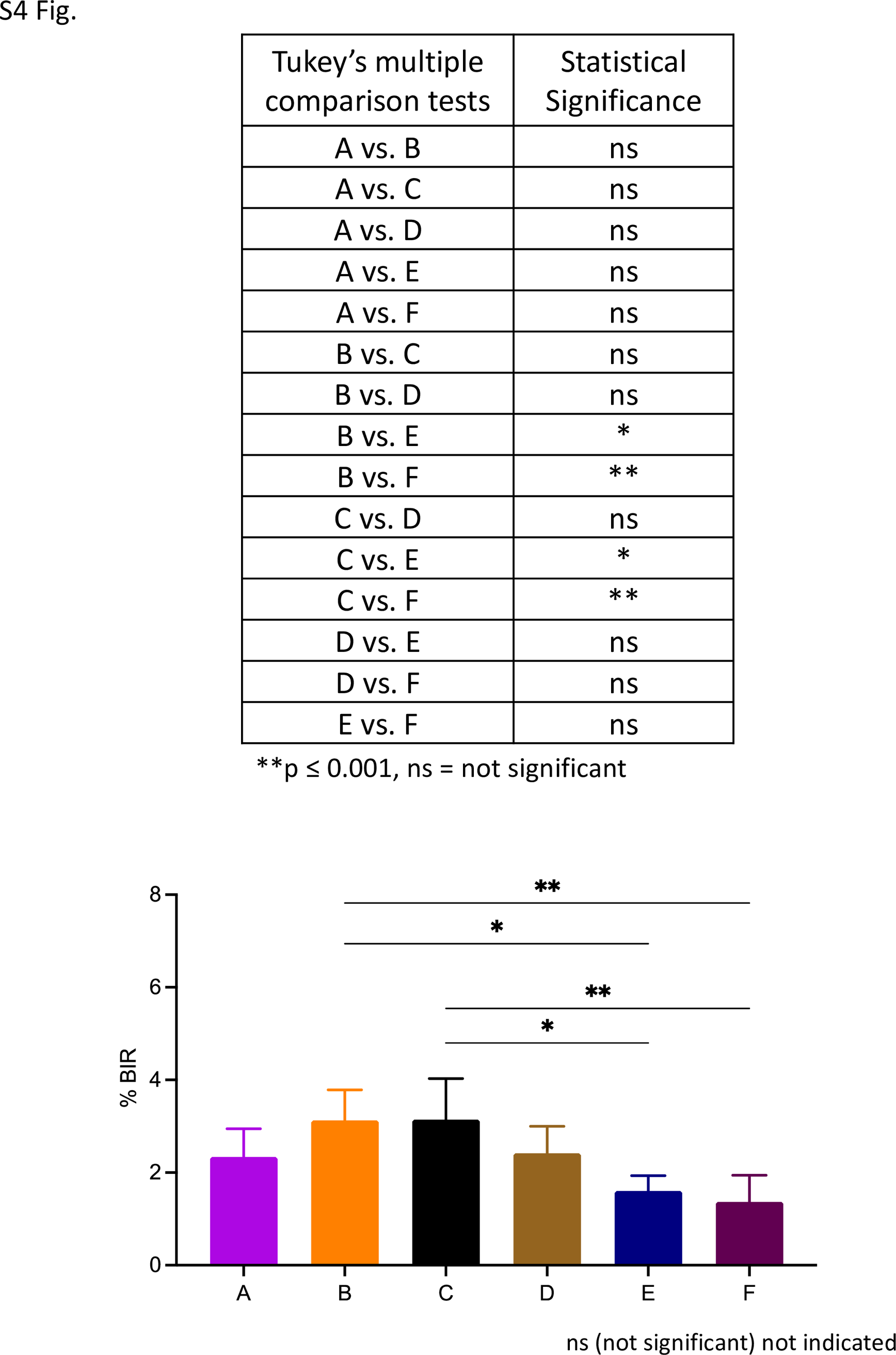

Supplement: S4 Fig — Donor template were compared among them to assess the statistical significance of different arrangements of clustered mismatches for BIR efficiency. Significance determined using a Tukey’s multiple comparison tests (GraphPad Prism 9). Error bars refer to standard deviation. ** p<0.001, ns = not significant. % BIR graph only indicated statistical significance and not included ns. (TIFF) [file pgen.1010056.s004.tiff]

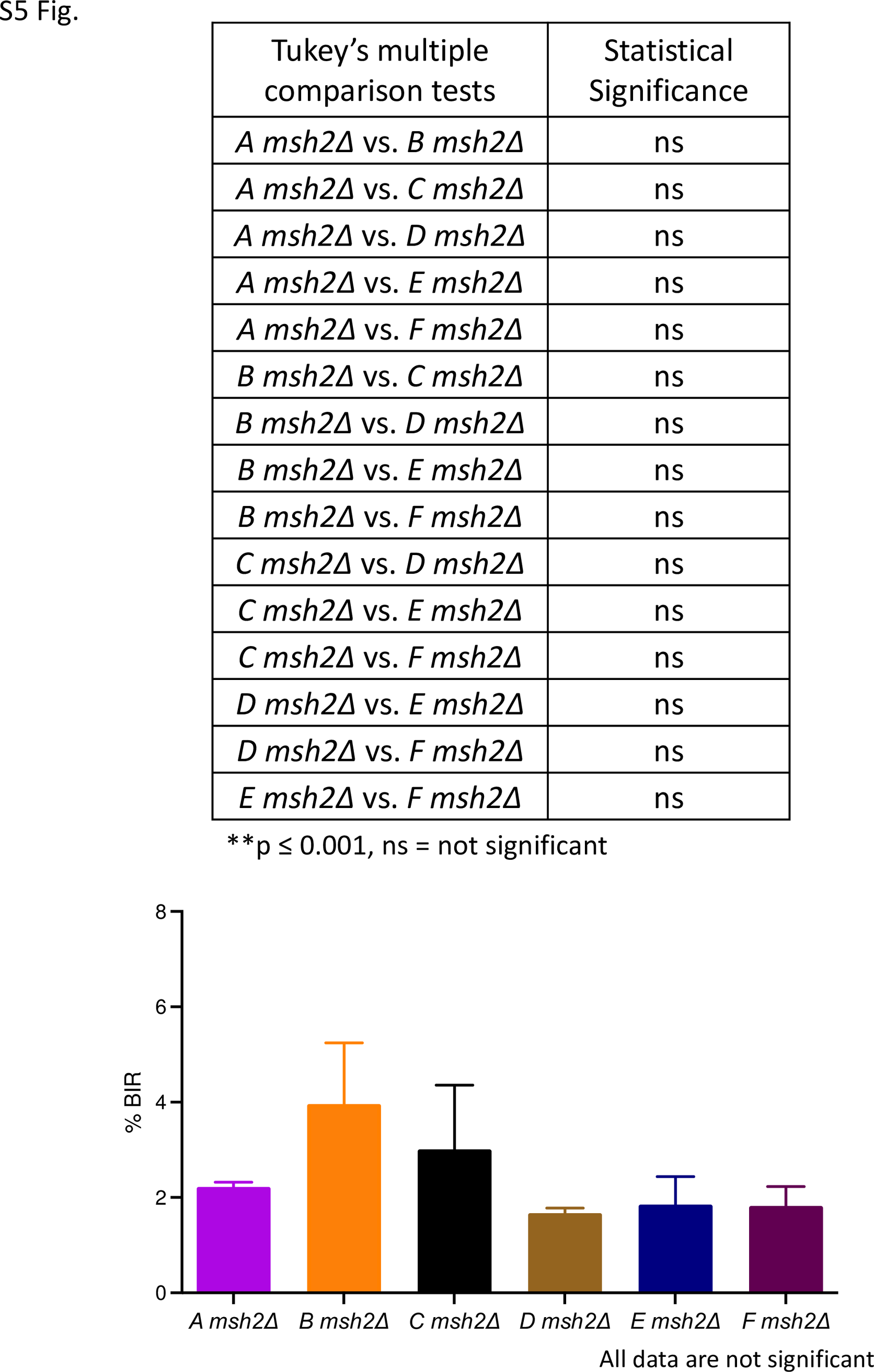

Supplement: S5 Fig — Each msh2Δ derivative of donor templates was compared between them to assess the statistical significance of different arrangements of clustered mismatches for BIR efficiency. Significance determined using a Tukey’s multiple comparison tests (GraphPad Prism 9). Error bars refer to standard deviation. ** p<0.001, ns = not significant. All data on % BIR graph for msh2Δ derivatives of donor templates were not significant when compared between them. (TIFF) [file pgen.1010056.s005.tiff]

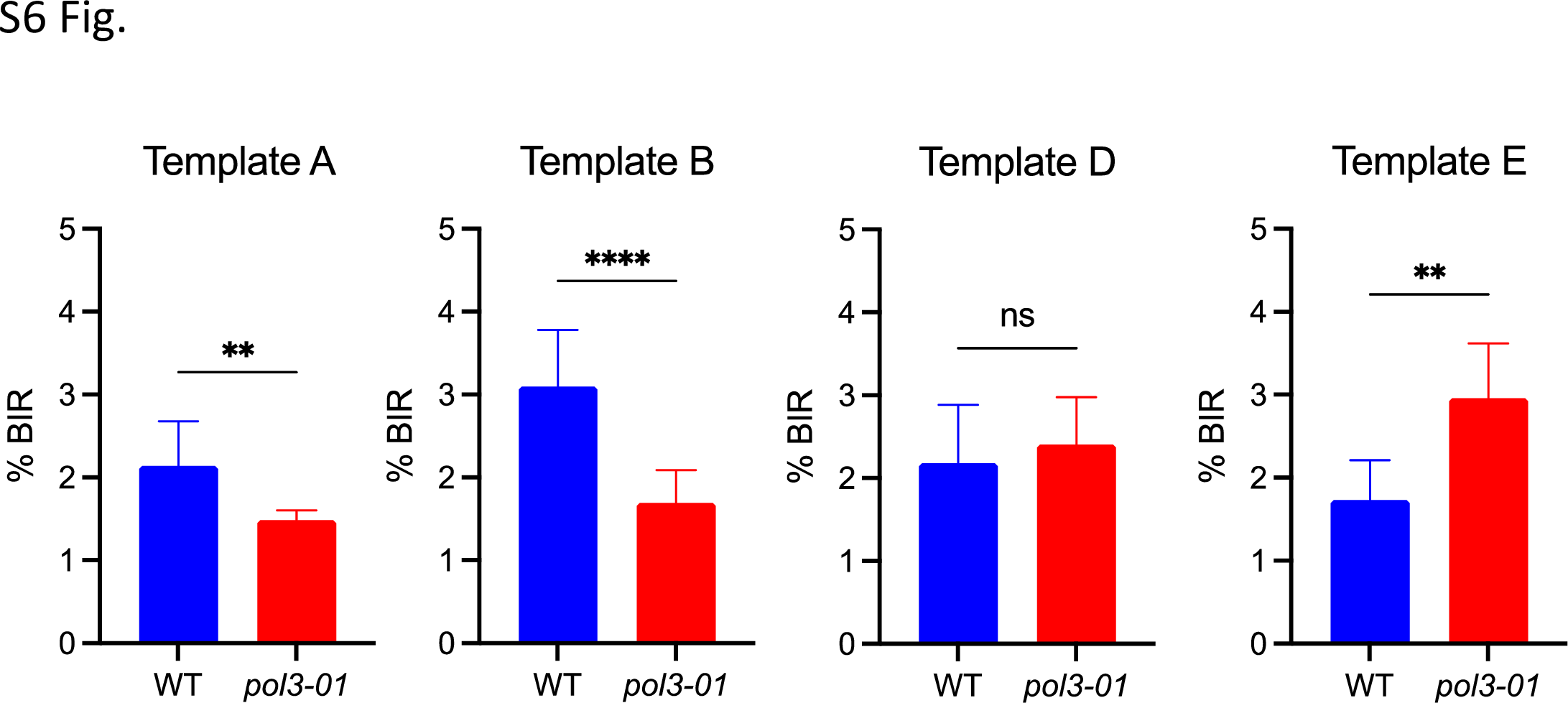

Supplement: S6 Fig — Percent BIR efficiency of donor templates A, B, D, and E with proofreading-defective DNA Polymerase δ mutant (pol3-01). Unpaired t-test with Welch’s correction was used to determine the p-value. Error bars indicate standard deviation. (TIFF) [file pgen.1010056.s006.tiff]

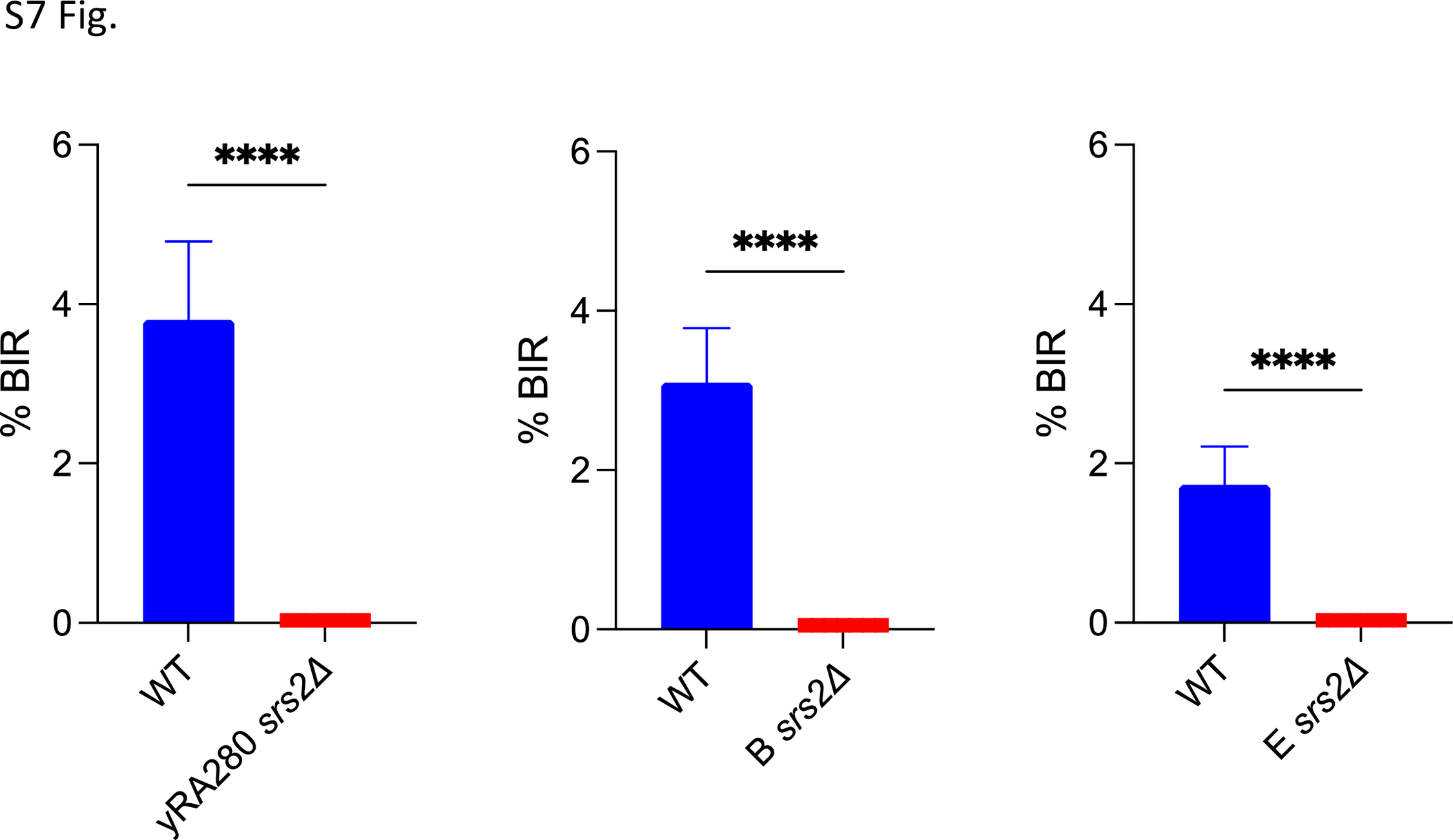

Supplement: S7 Fig — Percent BIR efficiency of yRA280 and donor templates B and E with srs2Δ. Unpaired t-test with Welch’s correction was used to determine the p-value. Error bars indicate standard deviation. (TIFF) [file pgen.1010056.s007.tiff]
