# Supplementary material for: Repair of mismatched templates during Rad51-dependent Break-Induced Replication
Source: PLoS Genet. 2022 Sep 2;18(9):e1010056. doi: 10.1371/journal.pgen.1010056 (PMC9477423; doi:10.1371/journal.pgen.1010056)
Supplement: S1 Table — (DOCX) [file pgen.1010056.s009.docx]

**S1 Table. Yeast strains used in this study**

| **Strain Name** | **Strain Genotype** | **Strain Description** |
| --- | --- | --- |
| yRA111 | *MAT*a::*DEL HOcs::hisG* *ura-3D851* *trp1-DEL63* *leu2DEL::KAN* *hmlDEL::hisG hmr::ADE3 ade3::GAL::HO* *can1::*UR intron_SD-HOcs-NAT | Donorless Strain  (Only contains the recipient) |
| yRA253 | *MAT*a::*DEL HOcs::hisG* *ura-3D851* *trp1-DEL63* *leu2DEL::KAN* *hmlDEL::hisG hmr::ADE3 ade3::GAL::HO* *can1DEL::*UR intron_SD*::HOcs::NAT,* intron-SA-*A3::TRP1* | Perfect homology  (No mismatch) |
| yRA280 | *MAT*a::*DEL HOcs::hisG* *ura-3D851* *trp1-DEL63* *leu2DEL::KAN* *hmlDEL::hisG hmr::ADE3 ade3::GAL::HO* *can1DEL::*UR intron_SD*::HOcs::NAT,* intron-SA-*A3::TRP1* | 10 mismatches  every 10^th^ bp |
| yRA321 | *MAT*a::*DEL HOcs::hisG* *ura-3D851* *trp1-DEL63* *leu2DEL::KAN* *hmlDEL::hisG hmr::ADE3 ade3::GAL::HO* *can1DEL::*UR intron_SD*::HOcs::NAT,* intron-SA-*A3::TRP1* | 18 mismatches  every 6^th^ bp |
| A | *MAT*a::*DEL HOcs::hisG* *ura-3D851* *trp1-DEL63* *leu2DEL::KAN* *hmlDEL::hisG hmr::ADE3 ade3::GAL::HO* *can1DEL::*UR intron_SD*::HOcs::NAT,* intron-SA-*A3::TRP1* | 10 mismatches  (5 mismatches on both 3’ and 5’ end, 52bp perfect homology in the middle) |
| B | *MAT*a::*DEL HOcs::hisG* *ura-3D851* *trp1-DEL63* *leu2DEL::KAN* *hmlDEL::hisG hmr::ADE3 ade3::GAL::HO* *can1DEL::*UR intron_SD*::HOcs::NAT,* intron-SA-*A3::TRP1* | 10 mismatches  (10 mismatches clustered on 3’end, 53bp perfect homology on 5’end) |
| C | *MAT*a::*DEL HOcs::hisG* *ura-3D851* *trp1-DEL63* *leu2DEL::KAN* *hmlDEL::hisG hmr::ADE3 ade3::GAL::HO* *can1DEL::*UR intron_SD*::HOcs::NAT,* intron-SA-*A3::TRP1* | 10 mismatches  (27bp perfect homology – 5 mismatches – 27bp perfect homology – 5 mismatches) |
| D | *MAT*a::*DEL HOcs::hisG* *ura-3D851* *trp1-DEL63* *leu2DEL::KAN* *hmlDEL::hisG hmr::ADE3 ade3::GAL::HO* *can1DEL::*UR intron_SD*::HOcs::NAT,* intron-SA-*A3::TRP1* | 10 mismatches  (10 mismatches clustered on 5’ end, 48bp perfect homology on 3’ end) |
| E | *MAT*a::*DEL HOcs::hisG* *ura-3D851* *trp1-DEL63* *leu2DEL::KAN* *hmlDEL::hisG hmr::ADE3 ade3::GAL::HO* *can1DEL::*UR intron_SD*::HOcs::NAT,* intron-SA-*A3::TRP1* | 10 mismatches  (27bp and 26bp perfect homology on both 3’ and 5’ end, 10 mismatches in the middle) |
| F | *MAT*a::*DEL HOcs::hisG* *ura-3D851* *trp1-DEL63* *leu2DEL::KAN* *hmlDEL::hisG hmr::ADE3 ade3::GAL::HO* *can1DEL::*UR intron_SD*::HOcs::NAT,* intron-SA-*A3::TRP1* | 10 mismatches  (5 mismatches – 27bp perfect homology – 5 mismatches – 26bp perfect homology) |
