# Supplementary material for: Repair of mismatched templates during Rad51-dependent Break-Induced Replication
Source: PLoS Genet. 2022 Sep 2;18(9):e1010056. doi: 10.1371/journal.pgen.1010056 (PMC9477423; doi:10.1371/journal.pgen.1010056)
Supplement: S2 Table — (DOCX) [file pgen.1010056.s010.docx]

**S2 Table. Recipient and donor sequences**

| Recipient sequence | Melting Temperature Tm (°C) | TAATATGGACTAAAGGAGGCTTTTCTGCAGGTCGATCTAAATAAATTCGTTTTCAA  TGATTAAAATAGCATAGTCGGGTTTTTCTTTTAGTTTCAGCTTTCCGCAACA |
| --- | --- | --- |
| Perfect homology donor  (yRA253) | 89.0 °C | TAATATGGACTAAAGGAGGCTTTTCTGCAGGTCGATCTAAATAAATTCGTTTTCA  ATGATTAAAATAGCATAGTCGGGTTTTTCTTTTAGTTTCAGCTTTCCGCAACA |
| Mismatches every 10^th^ bp  (yRA280) | 80.6 °C | TAATATGGtCTAAAGGAGcCTTTTCTGCtGGTCGATCTtAATAAATTCcTTTTCA  ATGtTTAAAATAGgATAGTCGGGaTTTTCTTTTtGTTTCAGCTaTCCGCAACA |
| Mismatches every 6^th^ bp  (yRA321) | 71.8 °C | aAATATcGACTAtAGGAGcCTTTTgTGCAGcTCGATgTAAATtAATTCcTTTTCtA  TGATaAAAATtGCATAcTCGGGaTTTTCaTTTAGaTTCAGgTTTCCcCAACA |
| Donor Template A | 83.5 °C | TAATAaGGACTcAAGGAcGCTTTgCTGCAaGTCGATCTAAATAAATTCGTTTTCA  ATGATTAAAATAGCATAGTCGGGTTTTgCTTTTtGTTTCgGCTTTaCGCAACc |
| Donor Template B | 82.8 °C | TAATATGGACTAAAGGAGGCTTTTCTGCAGGTCGATCTAAATAAATTCGTTTTgA  ATGAcTAAAAcAGCATgGTCGGcTTTTTgTTTTAaTTTCAtCTTTCgGCAACt |
| Donor Template C | 83.6 °C | TAATATGGACTAAAGGAGGCTTTTCTGgAGGTCaATCTAtATAAAaTCGTTcTCA  ATGATTAAAATAGCATAGTCGGGTgTTTCTaTTAGTcTCAGCgTTCCGtAACA |
| Donor Template D | 83.4 °C | TAATAcGGACTtAAGGAcGCTTTgCTGCAtGTCGAcCTAAAaAAATTtGTTTTgA  ATGAgTAAAATAGCATAGTCGGGTTTTTCTTTTAGTTTCAGCTTTCCGCAACA |
| Donor Template E | 82.0 °C | TAATATGGACTAAAGGAGGCTTTTCTGgAGGTCcATCTAgATAAAcTCGTTaTCA  ATaATTAAtATAGCcTAGTCtGGTTTgTCTTTTAGTTTCAGCTTTCCGCAACA |
| Donor Template F | 80.4 °C | TAATAaGGACTcAAGGAcGCTTTgCTGCAaGTCGATCTAAATAAATTCGTTTTCA  ATaATTAAtATAGCcTAGTCtGGTTTgTCTTTTAGTTTCAGCTTTCCGCAACA |
