# Supplementary material for: Repair of mismatched templates during Rad51-dependent Break-Induced Replication
Source: PLoS Genet. 2022 Sep 2;18(9):e1010056. doi: 10.1371/journal.pgen.1010056 (PMC9477423; doi:10.1371/journal.pgen.1010056)
Supplement: S3 Table — (DOCX) [file pgen.1010056.s011.docx]

**S3 Table. Primers used in this study**

| **Primer Name** | **Sequence** | **Primer Description** |
| --- | --- | --- |
| DG31 | GGAACGTGCTGCTACTCATC | Anneal to the start of URA3 allele to screen URA^+^ colonies |
| DG32 | TTGCTGGCCGCATCTTCTCA | Anneal to the end of URA3 allele to screen URA^+^ colonies |
| DG191 | CCCTTTCGTCTCGCGCGTTT | Amplify gBlock donor to build A-F strains |
| DG192 | GCTCTGTTATAAATAATACC | Amplify gBlock donor to build A-F strains (Reverse) |
| DG319 | ATGGGAACCATAAGCTGGACACATTTCTTGAGGTT  CTGATGCGGTATTTTCTCCTTACGC | Integrate constructs with divergent mismatch distribution downstream of FAU1 (Reverse) |
| DG320 | CGAGAAAGGCACTCTCTCACTGAAAAGTAATGAAA  TAATAAGGACTCAAGGACGCTTTGC | Integrate donor sequence from bRA29 plasmid with gBlock for A |
| DG321 | CGAGAAAGGCACTCTCTCACTGAAAAGTAATGAAA  TAATATGGACTAAAGGAGGCTTTTC | Integrate donor sequence from bRA29 plasmid with gBlock for E and B |
| DG322 | CGAGAAAGGCACTCTCTCACTGAAAAGTAATGAAA  TAATACGGACTTAAGGACGCTTTGC | Integrate donor sequence from bRA29 plasmid with gBlock for B |
| DG326 | AATGTGCTCCACCATCACAG | Anneal 798 bp upstream of FAU1 to confirm integration of divergent constructs |
| DG327 | CAGGCCTTTGATGGAATCAG | Anneal 314bp upstream of FAU1 to confirm integration of divergent construct |
| JC1 | CCAGCGAAGACTGTCGTATCGTTTT | gRNA1 to target FAU1 |
| JC2 | GATACGACAGTCTTCGCTGGGATCA | gRNA 2 to target FAU1 |
| JC14 | CCCTTTCGTCTCGCGCGTTTCGGTGATGACGGTGAAAACCTCTGACACATGCAGCTAATAAGGACTGAAG | Integrate donor sequence from bRA29 plasmid with gBlock for F |
| JC15 | TGCTCTGTTATAAATAATACCATTTGTTAGTAAAAATT  CGAGCTCGGTACCCGGGTGTTGCGGAAAGCTG | Integrate donor sequence from bRA29 plasmid with gBlock for F |
| JC16 | CCCTTTCGTCTCGCGCGTTTCGGTGATGACGGTGAAAACCTCTGACACATGCAGCTAATATGGACTAAAG | Integrate donor sequence from bRA29 plasmid with gBlock for C |
| JC17 | TGCTCTGTTATAAATAATACCATTTGTTAGTAAAAATT  CGAGCTCGGTACCCGGGTGTTACGGAACGCTG | Integrate donor sequence from bRA29 plasmid with gBlock for C (Reverse) |
