# Supplementary material for: Repair of mismatched templates during Rad51-dependent Break-Induced Replication
Source: PLoS Genet. 2022 Sep 2;18(9):e1010056. doi: 10.1371/journal.pgen.1010056 (PMC9477423; doi:10.1371/journal.pgen.1010056)
Supplement: S4 Table — (DOCX) [file pgen.1010056.s012.docx]

**Table 4. Plasmids used in this study**

| **Plasmid Name** | **Plasmid Description** |
| --- | --- |
| bRA25 | pRS314-based plasmid containing the 5’ sequences from the *URA3* gene (UR), an artificial split-intron with the splice-donor site (5′ SD) and the HO recognition site (HOcs) |
| bRA29 | pRS314-based (CEN4; TRP1) plasmid containing the 3’ splice-acceptor of the intron, the 3’ sequence from the *URA3* gene (A3), and the TRP 1 marker |
| pUC19_108 | pUC19-based plasmid containing the perfect homology donor (yRA253) sequence inserted at the SmaI cut site |
| Curmid | pUC19-based plasmid containing the 9.7 kbp SphI digest product from bacteriophage λ-DNA inserted at the SphI site |
