# Supplementary material for: Repair of mismatched templates during Rad51-dependent Break-Induced Replication
Source: PLoS Genet. 2022 Sep 2;18(9):e1010056. doi: 10.1371/journal.pgen.1010056 (PMC9477423; doi:10.1371/journal.pgen.1010056)
Supplement: S5 Table — (DOCX) [file pgen.1010056.s013.docx]

**S5 Table. Sequences of 90-nt ssDNA containing evenly-distributed mismatches**

| **Mismatch frequency** | **Sequence** |
| --- | --- |
| No mismatch | CGC TCC ATC GTT TCA CGG ACC TGC TGG GCA AAA ATT TCC TGA TAG TCG TCA CCG CGT TTT GCG CAC TCT TTC TCG TAG GTA CTC AGT CCG |
| Every 3^rd^ | gGC aCC tTC cTT aCA gGG tCC aGC aGG cCA tAA tTT aCC aGA aAG aCG aCA gCG gGT aTT cCG gAC aCT aTC aCG aAG cTA gTC tGT gCG |
| Every 5^th^ | gGC TCg ATC GaT TCA gGG ACg TGC TcG GCA tAA ATa TCC TcA TAG aCG TCt CCG CcT TTT cCG CAg TCT TaC TCG aAG GTt CTC AcT CCG |
| Every 6^th^ | gGC TCC tTC GTT aCA CGG tCC TGC aGG GCA tAA ATT aCC TGA aAG TCG aCA CCG gGT TTT cCG CAC aCT TTC aCG TAG cTA CTC tGT CCG |
| Every 7^th^ | CGC TCC tTC GTT TgA CGG ACg TGC TGG cCA AAA AaT TCC TGt TAG TCG aCA CCG CcT TTT GCc CAC TCT aTC TCG TtG GTA CTg AGT CCG |
| Every 8^th^ | CGg TCC ATC GaT TCA CGG tCC TGC TGc GCA AAA AaT TCC TGA aAG TCG TCt CCG CGT TaT GCG CAC aCT TTC TCc TAG GTA CaC AGT CCG |
| Every 9^th^ | gGC TCC ATC cTT TCA CGG tCC TGC TGG cCA AAA ATT aCC TGA TAG aCG TCA CCG gGT TTT GCG gAC TCT TTC aCG TAG GTA gTC AGT CCG |
| Every 12^th^ | CGC TCC tTC GTT TCA CGG tCC TGC TGG GCA tAA ATT TCC TGA aAG TCG TCA CCG gGT TTT GCG CAC aCT TTC TCG TAG cTA CTC AGT CCG |
| Every 15^th^ | gGC TCC ATC GTT TCA gGG ACC TGC TGG GCA tAA ATT TCC TGA TAG aCG TCA CCG CGT TTT cCG CAC TCT TTC TCG aAG GTA CTC AGT CCG |
| Every 30^th^ | gGC TCC ATC GTT TCA CGG ACC TGC TGG GCA tAA ATT TCC TGA TAG TCG TCA CCG CGT TTT cCG CAC TCT TTC TCG TAG GTA CTC AGT CCG |
