# Supplementary material for: Repair of mismatched templates during Rad51-dependent Break-Induced Replication
Source: PLoS Genet. 2022 Sep 2;18(9):e1010056. doi: 10.1371/journal.pgen.1010056 (PMC9477423; doi:10.1371/journal.pgen.1010056)
Supplement: Attachment — Submitted filename: review_Choi_et_al.pdf [file pgen.1010056.s014.pdf]

Data presented in the manuscript indicating that:

- stretches of homology at the 5' end may have unappreciated role for BIR efficiency at least as important (maybe in some cases even more) as homology at the 3' invading end
- during BIR polymerase δ mediates extensive resection even into perfectly homologous sequence

are both unexpected and interesting findings that possibly extend the conventional view of how this repair process is executed.
However, their description is vague, often imprecise or unclear, full of generalizations, and no hypothesis is provided how these findings could be incorporated into the model for BIR inferred from previous studies.

Abstract, Author summary

line 43
BIR efficiency was the same whether the mismatches were clustered at the 3' invading end or at the 5' end

But for all the templates examined where majority of mismatches were near 5' end (A, D, E, F) BIR efficiency was lower than for evenly spaced yRA280 control and for templates B, C with majority of mismatches closer to the 3'. For B, C BIR efficiency was higher, similar to yRA280 control.

Different relations still are apparent from comparisons with yRA321 control.

line 48
These observations enrich our understanding of the details of Rad51-mediated strand invasion

In what way? No hypothesis, model provided.

Introduction

line 86
...although even when every 6th base was mismatched, BIR occurred at 10% of the rate when the templates were identical [24]. Repair efficiency was significantly reduced but still significant when every 6th base was mismatched.

Unclear, in ref. 24 BIR efficiency is estimated as 13.8% for no MM; 0.8% for every 6th base MM (6% success relative to no MM); ~3.5% for every 10th base MM (~25% success relative to no MM).

line 92
The differences among these studies may reflect the longer (108-bp) substrates used in our in vivo studies or the agency of other recombination proteins not presented in the in vitro studies.

Including information on substrate length used in cited refs. 30 and 31 would help the reader to evaluate the Authors' argument.

line 99
We show that these different arrangements were in general less efficient than the evenly distributed mismatches, but exhibit significant differences that depend on the precise location of the mismatches.

Imprecise, effect depends on the reference strain, for yRA280 BIR is less efficient for 4 out of 6 arrangements, for yRA321 BIR is more efficient for different 4 out of 6 arrangements.

What is specifically meant by *significant differences*?

Results

line 130
BIR was reduced to about 44% in yRA280 compared to yRA253, and still occurred about 9% in yRA321 (Fig. 2B).

Confusing, given the values in Fig.1B Perhaps both values: %BIR as overall viability of Ura3+ cells completing BIR and %BIR as "% success" relative to value for no MM control could be given.

Fig. 2C, line 135
The efficiency of BIR for the substrate with all mismatches clustered at the 3' invading end of the DSB (Fig. 2C, donor template B) was similar to the reference yRA280, and was surprisingly more efficient than with a substrate with a 48 bp of perfect homology at the 3' end (Fig. 2C, donor template D).

Seems to be so but statistical significance for changes in BIR for different arrangements of clustered MM were calculated relative to even spaced controls. In order to assess the importance of different types of clustering for BIR efficiency, comparisons between them should be included, i.e. B (and C) with D, to validate the statement quoted above, but also D with E, F, etc.

line 141
...the efficiency of repair was not more impaired...

Understatement, in fact BIR efficiency was less impaired when the 3' invading end of the DSB was mismatched (B) than when distal 5' end of homologous sequence was mismatched (D), at least when compared to yRA321 (Fig.2C). As quoted by the Authors in line 135.

line 143
... but other substrates with 26 bp of perfect homology at the 3' end (Fig. 2C, donor templates E and F) yielded a significantly lower rate of successful recombination when compared to yRA280 (Fig. 2C).

E and F perform worse also when compared to yRA321 given that all other substrates (A,B,C,D) except E and F were more efficient at BIR than this even spaced control.

line 150
...but the correlation was shifted to a lower average value....

Imprecise, the Authors do not provide correlation values (slopes of the fitted lines relevant to correlations seem the same, not shifted). Average values of %BIR are lower for unevenly spaced MM arrangements, compared to yRA280 control, but note that the difference in %BIR is statistically significant for only 4 of them (Fig. 2C).

Fig. 3B

No description/discussion of these data in the text.

line 157
We created a set of msh2Δ derivatives of the set of templates and found that the differences between the evenly-spaced mismatches and the uneven cases were not suppressed (Fig. 3 and Fig. S2).

When one compares Fig. 2C and 5C (which is not referred to in the above description) it seems that these differences were suppressed for A and perhaps D,E,F (one star not three) for comparison with yRA280 as well as for A,C and D for comparison with yRA321.

However, the efficiency of BIR was generally reduced relative to wild type, even for the case of no mismatches...

All differences presented in Fig. S2 are marked as not significant. It is unclear what the Authors refer to as wild type? What do they compare with what?

....and the differences between yRA280 and yRA321 was no longer statistically significant (Fig. S2).

Statistical significance for comparison between yRA280*msh2Δ* and yRA321*msh2Δ* is not shown in Fig.S2 (as it is not shown for yRA280 and yRA321 here or in Fig.2B).

It would also be interesting to discuss the effect of *msh2Δ* on differences between B and D.

Fig. 4

Perhaps data on panels A and B could be combined. Precise number of sequenced clones for each construct could be given next to the legend, this would give the reader the idea of the resolution that can be achieved.

Is the difference between templates A and C in panel D meaningful?

Fig. 5

Removing MM beyond ~30nt becomes dependent on Msh2&Mlh1 so why only donor template A was used for comparison, and not template B and/or C? It would also be interesting to see if MM incorporation for templates E&F is affected in both mutant backgrounds (if resolution allows it).

line 189
There are no consistent differences between wild type, msh2Δ and mlh1Δ strains;

In what? MM incorporation or %BIR for new data with *mlh1Δ* that were not discussed before.

…the differences in assimilation most likely reflect the limited number of sequences used to measure mismatch assimilation.

The Authors should provide the number of sequences analysed and explain how that can influence the data presented in the figure.

Discussion

line 205
…the success of repair is primarily a function of the total number of base pairs that can be formed over the entire region.

True, given correlation with Tm, but still BIR efficiency for template D seems to be worse than for B. This is most unexpected and interesting result, and the Authors avoid discussing it. It would be interesting if they could hypothesize on the possible significance of such distal homology for efficiency/stability of pairing and repair, especially *in vivo* when Rad51 filament must discriminate between many potential homologous templates. Perhaps this could suggest the reason why *in vivo* longer regions of homology are required for BIR to take place, as shown in ref. 24.

Perhaps it is insignificant but the Authors could at least address the issue.

line 204
However, none of the arrangements of 10 mismatches with every 6th base spacing was as efficient as a template with 10 mismatches, spaced every 10 base pairs, although in two cases the difference was not statistically significant.

If the difference for two templates was not significant, then these two templates with clustered mismatches should be regarded as efficient as equally spaced control.

lines 207-215
This part lacks clarity and it is difficult to follow the Author's reasoning.

line 227
…deleting Msh2 led to an overall reduction in repair efficiency for both the evenly- and unevenly-spaced templates so that any differences became statistically insignificant….

Data shown in Fig. S2 demonstrate that differences between respective templates in *WT* and *msh2Δ* mutant backgrounds are not statistically significant so the Authors should not claim that deletion of *MSH2* caused reduction of repair. Why should it?

…. but a similar correlation with Tm persists.

It is impractical to assess the correlation for a set of data that don't differ significantly from each other (cf. Fig 5C).

It would be informative to the reader if the Authors explained in more detail how their findings might be relevant to the role of Pol δ in meiosis.
