# Supplementary material for: Repair of mismatched templates during Rad51-dependent Break-Induced Replication
Source: PLoS Genet. 2022 Sep 2;18(9):e1010056. doi: 10.1371/journal.pgen.1010056 (PMC9477423; doi:10.1371/journal.pgen.1010056)
Supplement: Attachment — Submitted filename: Response to Reviewers Comments_JC_BIR_2022_final_submit.docx [file pgen.1010056.s015.docx]

Revision and resubmission of the manuscript PGENETICS-D-22-00123

Thank you for giving us the opportunity to submit a revised draft of our manuscript, *Repair of Mismatched Templates during Rad51-dependent Break-Induced Replication*. We appreciate the careful review and insightful comments on our manuscript. We have carefully considered the comments and tried our best to address changes to reflect the feedback and suggestions provided by the reviewers. The point-by-point responses to comments and concerns from all reviewers are marked in blue.

We note that the paper has been amended by the addition of data from Eric Greene’s lab in which we have been able to resolve what appeared to be a very significant difference between *in vivo* and *in vitro* analysis of the effect of mismatches on recombination. Green and Sung’s labs had found – using relatively short DNA substrates – that a stable strand association (D-loop formation) required 8 consecutive base pairs, whereas our study of in vivo BIR, with a template of 108 bp) showed that even a substrate with only 5 consecutive base pairs (every 6^th^ mismatched) had significant levels of exchange. We now include data showing that in vitro D-loop assays with 90 or 108-bp are remarkably similar to the in vivo results. This is, in and of itself, a significant finding. But we still see that there are systematic differences between in vivo and in vitro results, as discussed in the revised MS.

**Response to Reviewer #1**

**Reviewer #1:** This manuscript will be of interest for researchers in the area of genome stability and mechanisms of homology-directed DNA repair. The study follows an earlier high-profile paper (Anand et al. 2017 Nature), where the Haber laboratory identified an astounding mismatch tolerance of Rad51-mediated break-induced replication. Here, the authors conduct careful analysis of additional mismatched constructs that test hypotheses that length and position of uninterrupted homology affect mismatch tolerance. The analyses are conducted in wild type and mismatch repair-deficient strains disabling mismatch recognition (msh2) and mismatch processing (mlh1). Sequence analysis of individual recombinants is used to assess the correction of the mispairs formed during strand invasion. The experiments are well designed and executed, but the results limit potential conclusions, and the work does not generate novel insights. A number of the conclusions appear overly generalized and do not consider the unusual properties of the system used here. In particular, the main conclusion that the exonuclease activity of DNA polymerase delta can chew back for 26 nt without mismatch cues is overstated and an alternative model of reiterative invasion and chew-back should be considered and experimentally tested.

Major comments:
**[Comment # 1]:** In the introduction (line 77 ff), the authors discuss the properties of their assay system and correctly point out that it is above the minimal homology requirement for recombination in yeast. However, the authors should also discuss the differences to system with longer homology and infinite homology, as it would be the case for allelic HR between sisters/homologs. In other words, the authors should acknowledge the limitations of the system sharing only 108 bp homology. Moreover, the authors assume that the D-loops in their study are uniform in structure and length, all invading with their 3’-end, although it is currently not possible to analyze these D-loop properties *in vivo*. Furthermore, the authors neglect to discuss discrepancies between their findings and other published studies. Guo et al. 2017 Mol Cell conducted a similar study with dramatically different conclusions as to the role of Pol delta in 3’ mismatch correction. A discussion of this study and other similar studies should be included to contextualize the findings presented in Choi et al. in the broader HR field.
**[Authors’ Response #1]:**

We did not mean to imply that strand invasion needed to start at the 3’ end, but the 3’ end has to be paired in order for DNA polymerase to extend the end. We have re-written the text accordingly and have provided several references showing that strand invasion can occur in vitro without a free 3’ end.

**[Comment # 2]:** Figure 2 and lines 82, 83, and throughout: “approximately 14% of cells successfully repaired a DSB when the homologous region is 108 bp”, this value differs from what is shown in Figure 2B, which shows ~8-9% of cells repairing by BIR. The text should be corrected to reflect the data reported in the figure.

**[Authors’ Response #2]:** We quoted the original data, as published, in the introduction; but it is the case – as the reviewer notes – that our more recent data in this paper consistently shows a lower level of repair than the original data, although the reductions seen for mismatched substrates were proportionally reduced. We have acknowledged this difference in a footnote stating that the same assay, done by different people at a different time, with who knows what changes to media or H2O or some other unknown, yields consistently lower outcomes).

**[Comment # 3]:** Figure 2 and lines 87-90, 129, 130: “Repair efficiency was significantly reduced but still significant when every 6th base was mismatched”; the authors should reference Figure 2B. Furthermore, it is debatable whether a repair event observed in only 1-2% of the population is “significant.” The authors should give the actual percent of cells completing repair. Considering repair is almost negligible in this system when every 6th bp is mismatched, it is misleading to claim that their in vivo results differ from results obtained by in vitro studies.

**[Authors’ Response #3]:** We agree with the reviewer and the text in lines 87-90, 129, 130 now refer to Figure 2B as the reviewer suggested. % viability completing repair (the actual percent of DSB survivors) are added as part of Figure 2B.

But we disagree with the reviewer about the significance of repair at a 1-2% level. We could easily measure repair down to less than 0.1% and these differences are indeed statistically significant: this value is still 10-20% of the efficiency with perfectly matched substrates. Mutation assays sometimes report changes from 10^-7^ to 10^-8^, and these measurements, done properly, are also significant.

As the reviewer notes, our results appeared to be in sharp contrast to the in vitro studies of Greene and Sung, where recombination apparently required 8 consecutive bases to pair (i.e. the limit would be with every 9^th^ bp mismatched. In new data added to this MS we resolve this apparent contradiction, showing that D-loop assays with substrates of 90 or 108 bp (comparable to the 108-bp assays we have used in vivo, the results are strikingly comparable (Fig. 4 in the revised paper). So it is substrate length rather than the absence of some key repair factors *in vitro* that accounts for the apparent difference. This is a very important finding.

**[Comment # 4]:** Figure 2 and Line 101: “but exhibit significant differences that depend on the precise location of the mismatches”; not all the differences are significant, and the authors fail to interpret their findings in a way that makes a convincing argument that the position of the mismatches influences the outcome in a predictable or logical manner. The results simply do not lend themselves to making significant conclusions about position of uninterrupted homology.
**[Authors’ Response #4]:** As suggested by the reviewer, we have revised the text to note that 3 of the 4 donors with 3’ ends perfectly matched to the invading strand have statistically significantly lower repair than the evenly spaced control, whereas constructs with mismatches clustered at the 3’ end were not different from the control.

**[Comment # 5]:** Figure 3 and lines 157-161: msh2 clearly effects [sic] the efficiency of BIR in a substrate-dependent manner according to Figures 3 and S2. The perfectly matched donor (yRA253) shows decreased BIR in the msh2 background. However, the donor with every 6th bp mismatched (yRA321) shows somewhat increased BIR in the msh2 background. Furthermore, the substrates with 3’ mismatches (‘B’), with mismatches in the middle of the donor (‘E’), and with mismatches at the 5’ end and in the middle of the donor (‘F’) also have slightly increased BIR in the msh2 background. In addition, the slope of the line in Figure 3B flattens relative to Figure 3A. This indicates that while msh2 does not fully suppress the differences between the evenly-spaced mismatches and the unevenly-spaces mismatches, at least some of the differences are partially suppressed. The authors need to revise these lines to better fit their findings.
**[Authors’ Response #5]:** We agree that “*msh2* does not fully suppress the differences between the evenly spaced and unevenly spaced mismatches" in that they continue to lie as a group below the evenly spaced control in Fig. 3. However – and we failed to make this sufficiently clear in the prior version - Fig. S2 shows clearly that there are no statistically significant changes in BIR efficiency for any given substrate with or without Msh2, using an unpaired t-test with Welch’s correction. Thus there is some other factor at work, as we had stated.

**[Comment # 6]:** Figures 4, 5 and Lines 108-111, 166-168, 232, 233: The authors could have made this point more convincingly by using the pol3-5DV mutant, as in Anand et al. 2017 and Guo et al. 2017 Mol Cell. Moreover, though the authors’ results on Pol delta’s proofreading activity differ significantly from the findings in Guo et al., the authors do not cite or discuss this paper in the text.
The effects of msh2 and mhl1 demonstrated in Figure 5 are neither acknowledged nor discussed. Lines 189, 190 should be rephrased and expanded.

**[Authors’ Response #6]:** Data from Gordenin and Burgers’ lab showed that *pol3-DV* and *pol3-01* were equivalently defective in 3’ to 5’ excision of mismatched ends (Jin et al., 2005 now referenced). We note that Anand et al. 2017 also used *pol3-01* and not *pol3-DV*. We now have directly confirmed that *pol3-01* affects repair similarly with unevenly spaced mismatches (Figure 6).

However, we did stumble on something unexpected. While *pol3-01* almost completely blocks assimilation of mismatches into the BIR product for sites 5 or more bases from the 3’ end, the incorporation of a mismatch at the first base was surprisingly unaffected. We had not previously tested a case with the first base mismatched. We do not know whether this reflects a residual activity of Pol3-01 (but in vitro assays showed no activity in removing a terminal mismatched base) or the existence of another 3’ to 5’ exonuclease activity limited to the very end.

In the revised MS, the effects of *msh2* and *mlh1* in Fig. 7 are acknowledged and further discussed in the text:

To confirm that assimilation of mismatches was independent of the Msh2/Mlh1-dependent mismatch repair system, we examined repair in the two reference strains, yRA280 and yRA321, as well as in donor template A (Fig. 7A and 7B). In each case, cells lacking MLH1 or MSH2 still can extend and correct mismatches >30 bp from the 3’ invading end of the DSB (Fig. 7A and 7B). The mismatch assimilation of *msh2Δ* derivatives of remaining donor templates (templates B through F) with uneven distribution also did not proceed through Msh2-dependent mismatch repair (Fig. S3).

**[Comment # 7]:** The major conclusion of the manuscript is that DNA polymerase delta exonuclease can chew back 26 nt with mismatch clues. This conclusion is not well supported by the analysis. Can the authors exclude alternative mechanisms or models? Is it possible that 3’-flappases like Rad1-Rad10 or Mus810-Mms4 play a role? Is it possible that he results are a reflection of reiterative cycles of invasion, chew-back and D-loop dissolution? This would be consistent with the expected instability of the short D-loop, the evidence for reiterative invasion cycles and the known proofreading window for Pol delta exo, which is significantly smaller than 26 nt. Moreover, this would be more consistent with the previous analysis by Guo et al. This possibility should be explored using mutants that limit D-loop dissolution (srs2, mph1, sgs1), and the results may drastically change the major conclusion.
**[Authors’ Response #7]:**

We note that in vitro studies of 5’ to 3’ resection of a labelled oligo bound to its complement can be resected extensively, as shown in Fig. from Jin et al, 2003 (below), as we now note in the text.

Nevertheless, we agree with the reviewer that there could be cycles of 3’ resection rather than a single event and have made this clear in the text. We don’t know how to test that, but we now include data ruling out the role of Rad1-Rad10 and Mph1-Srs2.

Additional comments:
**[Comment # 8]:** Figure 1, Table 1: Is pairing possible between the UR of the assay system and ura-3D851, also present in these strains? This could significantly influence the interpretation of the results. Even if there is no homology, the authors may want to state this clearly.

**[Authors’ Response #8]:** Although the ORF is deleted in ura3D851, the promoter region is still present, so there is 200 bp of remaining homology that might compete for a recombination event, although this would never be Ura3^+^. Such outcomes are apparently not viable (i.e. when mismatches prevent the BIR event we assay, there are no survivors other than NHEJ events at the HO cleavage site). If anything, we would expect that such a competition would reduce the usage of the highly mismatched donor sequences (that is, we would underestimate their usage). In any case we now note that such a competition is possible.

**[Comment # 9]:** Lines 54-57: NHEJ and alt-EJ are generally considered distinct pathways for DNA damage repair/tolerance.
**[Authors’ Response #9]:** we have made the clarification, and apologize for mis-stating something they we well know (having identified MMEJ in yeast).

**[Comment # 10]:** Lines 59, 60: Single-strand annealing (SSA) is a homology-directed repair pathway but not typically considered an HR sub-pathway. The discussion of DNA damage repair/tolerance pathways and HR sub-pathways could be re-written and expanded to include more relevant details.
**[Authors’ Response #10]:** We do not think it is necessary to provide a full catalogue of all possible HR events, since we do not deal with them at all in this paper. We do refer to several reviews where such events are discussed in detail.

**[Comment # 11]:** Lines 64-72: The description of BIR includes certain very specific details while glossing over other key steps (e.g. Rad51 mediators and accessory factors, Pol32 requirement). The description of BIR could be revised to include only the steps that are relevant to understanding the findings presented in this paper, and should be congruent with the diagram in Figure S1 (e.g. Rad52 is shown in the Figure but not discussed in the text).

**[Authors’ Response #11]:** We think it is reasonable to note BIR’s particular features, and we note in the legend to Fig. S1 that this diagram does not include all of the relevant steps in BIR. .

**[Comment # 12]:** Lines 78, 79: Both ends of the break do not necessarily interact with the donor during SDSA,
**[Authors’ Response #12]:** Agreed. Second end-capture involves an interaction of the second end with a new copy of the donor sequence. Wording has been changed.

**[Comment # 13]:** Line 111: “There was little effect when Msh2/Mlh1-dependent mismatch repair was ablated”; the results presented in Figure 3 indicate that there is an effect of msh2.

**[Authors’ Response #13]:** The effect of Msh2/Mlh1-dependent mismatch repair was seen in previous study (Anand et al, 2017), reducing the extent but not the overall frequency of assimilation of mismatches. We found here that while BIR efficiency is somewhat reduced in the absence of Msh2, mismatch assimilation of unevenly spaced templates near the 3’ end was not reduced and generally correction does not proceed through the general Msh2/Mlh1-dependend mismatch repair system.

As seen in Fig. 3B, their data points for the 6 unevenly-spaced templates were in close proximity to yRA280 *msh2Δ* background (Fig. 3B), which is consistent with nonsignificant statistical difference between yRA280 *msh2Δ* and *msh2Δ* derivatives of all unevenly-spaced templates (Fig. 5C, top graph).

**[Comment # 14]:** Lines 130, 131: “BIR…still occurred about 9% in yRA321”, this makes it sound as though 9% of the population completes repair by BIR with the yRA321 substrate, when in fact BIR is reduced to 9% of the level observed in yRA280. 9% repair by BIR would be a significant level of repair, but 1-2% repair is not necessarily significant. The authors need to clarify their results so as not to mislead the reader.

**[Authors’ Response #14]:** We agree with the reviewer that this statement need a clarification. The revised text reads as follows on the manuscript: We confirmed that strain yRA280 (with every 10th base pair mismatched) and yRA321 (with every 6th base pair mismatched) had reduced, but significant levels of repair compared to yRA253 (a fully homologous donor) (Fig. 2B).

**[Comment # 15]:** Lines 168,169: The authors should cite Guo et al. 2017 Mol Cell.
**[Authors’ Response #15]:** We apologize for not having done so previously and have done so now.

**[Comment # 16]:** Lines 225-228: “Indeed, deleting Msh2 led to an overall reduction in repair efficiency for both the evenly- and unevenly-spaced templates,” this statement is not quite correct based on their own results in Figures 3B and S2. BIR efficiency increased in the msh2 background for some of the mismatched donors, whether the donors had evenly spaced mismatches (yRA321) or unevenly spaced mismatches (‘A’, ‘E’, and ‘F’).
**[Authors’ Response #16]:** Although none of the pairwise comparisons between WT and *msh2*Δ for individual templates were statistically significantly different (Fig. S5), the differences between the WT and *msh2*Δ data as a whole were significant by a Mann-Whitney test (p = 0.004). We conclude that there is some other factor beyond the mismatch repair machinery that causes the unevenly-distributed mismatches to be less successful in BIR than the evenly-distributed controls.

**[Comment # 17]:** Figures 2, 3: Why is the color indicating construct ‘A’ different between figures? The color representing ‘A’ should be consistent.

**[Authors’ Response #17]:** The color representing donor template A for both Fig. 2 and Fig. 3 are now the same and consistent.

**[Comment # 18]:** Table 1: Generally, ‘::’ denotes that a gene has been deleted and replaced with another marker. Thus, while I infer that “can1DEL::UR intron_SD::HOcs::NAT” is the site of the DSB in their assay system, it might be more appropriate to write this as “can1::UR intron-SD-HOcs-NAT”. This is especially confusing considering another maker present in these strains is HOcs::hisG. In this case, I infer that the HOcs at its native locus has been deleted, but given the annotation of the assay system HOcs, it is unclear whether this is the case.

**[Authors’ Response #18]:** The reviewer is correct, and we have revised all genotypes in more appropriate way as suggested by the reviewer.

**Response to Reviewer #2**

**Reviewer #2:** Data presented in the manuscript indicating that:
• stretches of homology at the 5’ end may have unappreciated role for BIR efficiency at least as important (maybe in some cases even more) as homology at the 3’ invading end
• during BIR polymerase δ mediates extensive resection even into perfectly homologous sequence are both unexpected and interesting findings that possibly extend the conventional view of how this repair process is executed.
However, their description is vague, often imprecise or unclear, full of generalizations, and no hypothesis is provided how these findings could be incorporated into the model for BIR inferred from previous studies.

**Abstract, Author summary**

**[Comment 1]: line 43** *BIR efficiency was the same whether the mismatches were clustered at the 3’ invading end or at the 5’ end*

But for all the templates examined where majority of mismatches were near 5’ end (A, D, E, F) BIR efficiency was lower than for evenly spaced yRA280 control and for templates B, C with majority of mismatches closer to the 3’. For B, C BIR efficiency was higher, similar to yRA280 control. Different relations still are apparent from comparisons with yRA321 control.

**[Authors’ Response #1]:** We agree that the data show in at least 3 of 4 cases that having perfectly matched sequences near the 3’ end actually resulted in a significantly lower rate of BIR and we have changed the text accordingly.

Even though yRA280/yRA321 and all the templates contain the same number of mismatches with the same spacing between the mismatches (10 mismatches and 6-bp spacing between mismatches), their unevenly spaced mismatch distribution along the donor template has demonstrated that the position of the mismatches influences the repair efficiency depending on whether the majority of mismatches are unevenly clustered near 5’ end versus (Template A, D, E, and F) at near the 3’ invading end (Template B and C).

**[Comment 2]: line 48** *These observations enrich our understanding of the details of Rad51-mediated strand invasion*

In what way? No hypothesis, model provided.

**[Authors’ Response #2]:** We have removed this statement and focused instead a summary based on the inclusion of the data on *in vitro* D-loop assay.

**Introduction**

**[Comment 3]: line 86** ...*although even when every 6th base was mismatched, BIR occurred at 10% of the rate when the templates were identical [24]. Repair efficiency was significantly reduced but still significant when every 6th base was mismatched.*

Unclear, in ref. 24 BIR efficiency is estimated as 13.8% for no MM; 0.8% for every 6th base MM (6% success relative to no MM); ~3.5% for every 10th base MM (~25% success relative to no MM).

**[Authors’ Response #3]:** We have corrected the text to reflect the data reported in Figure 2B.

**[Comment 4]: line 92** *The differences among these studies may reflect the longer (108-bp) substrates used in our in vivo studies or the agency of other recombination proteins not presented in the in vitro studies.*

Including information on substrate length used in cited refs. 30 and 31 would help the reader to evaluate the Authors’ argument.

**[Authors’ Response #4]:** We have expanded this point by the inclusion of the new data on D-loop assays using 90 and 108-nt DNA strands. But to the referee’s point: Reference 30 uses 20-bp substrates containing different numbers of distributed matches and mismatches. Reference 31 uses 70-bp substrates containing an internal 8- to 15-nt tract of microhomology flanked by nonhomologous sequence. Therefore, both *in vitro* studies use significantly shorter length of substrates compared to our *in vivo* work. Also, reference 31 use substrates are flanked by nonhomologous sequence whereas our substrates have no additional nonhomologous sequences at the 3’ end (mentioned in the introduction). We added the information on substrate length used in 2 references as part of the text in the manuscript as suggested by the reviewer.

**[Comment 5]: line 99** *We show that these different arrangements were in general less efficient than the evenly distributed mismatches but exhibit significant differences that depend on the precise location of the mismatches.*

Imprecise, effect depends on the reference strain, for yRA280 BIR is less efficient for 4 out of 6 arrangements, for yRA321 BIR is more efficient for different 4 out of 6 arrangements.

What is specifically meant by *significant differences*?

**[Authors’ Response #5]:** Significant differences are those judged by appropriate statistical tests to be statistically significant. We have tried to make this clear throughout.

**Results**

**[Comment 6]: line 130** *BIR was reduced to about 44% in yRA280 compared to yRA253, and still occurred about 9% in yRA321 (Fig. 2B).*

Confusing, given the values in Fig.1B Perhaps both values: %BIR as overall viability of Ura3+ cells completing BIR and %BIR as “% success” relative to value for no MM control could be given.

**[Authors’ Response #6]:** We agree with the reviewer that this statement need a clarification. As suggested by the reviewer, we now added an overall % viability of DSB survivors and % BIR of Ura3^+^ cells completing BIR were both given in Fig. 2B.

**[Comment 7]: Fig. 2C, line 135** *The efficiency of BIR for the substrate with all mismatches clustered at the 3’ invading end of the DSB (Fig. 2C, donor template B) was similar to the reference yRA280, and was surprisingly more efficient than with a substrate with a 48 bp of perfect homology at the 3’ end (Fig. 2C, donor template D).*

Seems to be so but statistical significance for changes in BIR for different arrangements of clustered MM were calculated relative to even spaced controls. In order to assess the importance of different types of clustering for BIR efficiency, comparisons between them should be included, i.e. B (and C) with D, to validate the statement quoted above, but also D with E, F, etc.

**[Authors’ Response #7]:** As suggested, all donor templates were compared between each other to show statistical significance for change in BIR for different arrangements of clustered mismatches to validate the original statement. Ordinary one-way ANOVA multiple comparison test was used to find the statistical significance and the table of comparisons has been included in Fig. S4. As shown in Fig. S4, the comparison between B or C with D as well as D with E or F were found to be non-significant.

As a result of these comparisons, the text is rephrased as:

When compared to yRA280, donor template B, with all mismatches clustered at the 3’ invading end of the DSB (Fig. 2C) was as efficient as yRA280. In contrast, a substrate with a 48 bp of perfect homology at the 3’ end (Fig. 2C, donor template D) was statistically significantly less efficient than yRA280. Thus, although the 3’ end must be synapsed with the donor to allow DNA polymerase δ to initiate new DNA synthesis at the 3’ end, BIR efficiency was less impaired when the 3’ invading end of the DSB was mismatched (donor template B) than when distal 5’ end of homologous sequence was mismatched (donor template D). However, among all 6 templates there was no clear correlation between the location of the mismatches and their efficiency of usage (Fig. S4). Moreover, donor templates E and F, with 26 bp perfect homology at the 3’ end performed worse than the four other substrates.

**[Comment 8]: line 141** *...the efficiency of repair was not more impaired...*

Understatement, in fact BIR efficiency was less impaired when the 3’ invading end of the DSB was mismatched (B) than when distal 5’ end of homologous sequence was mismatched (D), at least when compared to yRA321 (Fig.2C). As quoted by the Authors in line 135.

**[Authors’ Response #8]:** The text is rephrased as: BIR efficiency was less impaired when the 3’ invading end of the DSB was mismatched (donor template B) than when distal 5’ end of homologous sequence was mismatched (donor template D). However, among all 6 templates there was no clear correlation between the location of the mismatches and their efficiency of usage (Fig. S4). Moreover, donor templates E and F, with 26 bp perfect homology at the 3’ end performed worse than the four other substrates.

**[Comment 9]: line 143** *... but other substrates with 26 bp of perfect homology at the 3’ end (Fig. 2C, donor templates E and F) yielded a significantly lower rate of successful recombination when compared to yRA280 (Fig. 2C).* E and F perform worse also when compared to yRA321 given that all other substrates (A,B,C,D) except E and F were more efficient at BIR than this even spaced control.

**[Authors’ Response #9]:** As suggested, we added additional text to reflect comments: Moreover, donor templates E and F, with 26 bp perfect homology at the 3’ end, performed worse than the four other substrates.

**[Comment 10]: line 150** *...but the correlation was shifted to a lower average value....*

Imprecise, the Authors do not provide correlation values (slopes of the fitted lines relevant to correlations seem the same, not shifted). Average values of %BIR are lower for unevenly spaced MM arrangements, compared to yRA280 control, but note that the difference in %BIR is statistically significant for only 4 of them (Fig. 2C).

**[Authors’ Response 10]:** The graphing details and correlations for the lines in Fig. 3 are now presented.

**[Comment 11]: Fig. 3B** No description/discussion of these data in the text.

**[Authors’ Response #11]:** We have added text:  In general, the efficiency of BIR was lower in the absence of Msh2 and there was an apparently lower slope of the relation between BIR efficiency and the Tm of the sequence, both for the three controls (y = 0.044x - 2.984 compared to y = 0.053x - 3.737) and for the 6 unevenly-distributed cases (y = 0.036x - 2.548 versus y = 0.053x - 4.079) (Fig. 3B).  Again, the six mismatched cases appeared to lie on a separate line, with a slope similar to that for the evenly-distributed controls. Although none of the pairwise comparisons between WT and *msh2*Δ for individual templates were statistically significantly different (Fig. S5), the differences between the WT and *msh2*Δ data as a whole were significant by a Mann-Whitney test (p = 0.004).

**[Comment 12]: line 157** *We created a set of msh2Δ derivatives of the set of templates and found that the differences between the evenly-spaced mismatches and the uneven cases were not suppressed (Fig. 3 and Fig. S2).*

When one compares Fig. 2C and 5C (which is not referred to in the above description) it seems that these differences were suppressed for A and perhaps D,E,F (one star not three) for comparison with yRA280 as well as for A,C and D for comparison with yRA321.

**[Authors’ Response #12]:** We do not distinguish among stars. The data are either at least p <0.05 or they are not. However, we made some modifications on finding a more appropriate test that fit for statistical significance for these data. For Fig. S5, we used a Tukey’s multiple comparison tests to compare between *msh2Δ* derivatives of the set of templates for uneven cases. However, a Mann-Whitney test shows that msh2 and WT data are significantly different. We remain convinced that *msh2* does not suppress the difference between the 6 unevenly spaced cases and the evenly-spaced references.

**[Comment 13]:** *However, the efficiency of BIR was generally reduced relative to wild type, even for the case of no mismatches...*

All differences presented in Fig. S2 are marked as not significant. It is unclear what the Authors refer to as wild type? What do they compare with what?

**[Authors’ Response #13]:** We have deleted this statement.

**[Comment 14]:** *....and the differences between yRA280 and yRA321 was no longer statistically significant (Fig. S2).*

Statistical significance for comparison between yRA280*msh2*Δ and yRA321*msh2*Δ is not shown in Fig.S2 (as it is not shown for yRA280 and yRA321 here or in Fig.2B).

**[Authors’ Response #14]:** The author is correct that the statistical significance for comparing yRA280 *msh2*Δ and yRA321 *msh2*Δ is not shown.

**[Comment 15]:** It would also be interesting to discuss the effect of *msh2*Δ on differences between B and D.

**[Authors’ Response #15]:** The differences between wild-type template B and D were not statistically significant and *msh2Δ* did not have any effect on the differences (Fig. S4 and S5).

**[Comment 16]: Fig. 4** Perhaps data on panels A and B could be combined. Precise number of sequenced clones for each construct could be given next to the legend, this would give the reader the idea of the resolution that can be achieved.

Is the difference between templates A and C in panel D meaningful?

**[Authors’ Response #16]:** We decided to put panels A and B separately because this way allows referencing them separately in the text easier for the reader when referring back to the figure for each template. As suggested, we put the number of sequences clones for all templates in the legend. There is no statistically significant difference between templates A and C in panel D.

**[Comment 17]: Fig. 5** Removing MM beyond ~30nt becomes dependent on Msh2&Mlh1 so why only donor template A was used for comparison, and not template B and/or C? It would also be interesting to see if MM incorporation for templates E&F is affected in both mutant backgrounds (if resolution allows it).

**[Authors’ Response #17]:** As suggested, *msh2Δ* derivatives of all donor templates were made and used for comparison. Their % BIR efficiency and % MM incorporation for all donor templates is included now as Fig. S3. Msh2 deletion background had no effect on all donor templates, which is consistent with the observation seen in the preexisting data for yRA280, yRA321 and donor template A. Both the information and the description suggested was added in Figs. S5 and as the result section of the manuscript.

**[Comment 18]: line 189** *There are no consistent differences between wild type, msh2*Δ *and mlh1*Δ *strains;*
In what? MM incorporation or %BIR for new data with *mlh1*Δ that were not discussed before.

**[Authors’ Response #18]:** There is no significance difference in % BIR between wild type, *msh2Δ* and *mlh1Δ* strains for yRA280, yRA321 and donor template A as observed in Fig. 7A and 7B. Also, their mismatch incorporation pattern observed in the same figure for both *msh2Δ* and *mlh1Δ* compared to their wild type has no significant differences. Further experiments are done on *msh2Δ* derivatives of all remaining donor templates with uneven mismatch distribution as shown in Fig. S3.

**[Comment 19]:** *...the differences in assimilation most likely reflect the limited number of sequences used to measure mismatch assimilation.*

The Authors should provide the number of sequences analyzed and explain how that can influence the data presented in the figure.

**[Authors’ Response #19]:** As suggested, the number of analyzed sequences are now all indicated in the legend of the graphs and in the figure descriptions. We performed a minimum of at least three measurements for the assays and analyzed a minimum of 40 independent DNA sequences of individual clones resulting from several assays for all % mismatch incorporation data presented in the paper.

**Discussion**

**[Comment 20]: line 205** *...the success of repair is primarily a function of the total number of base pairs that can be formed over the entire region.*

True, given correlation with Tm, but still BIR efficiency for template D seems to be worse than for B. This is most unexpected and interesting result, and the Authors avoid discussing it. It would be interesting if they could hypothesize on the possible significance of such distal homology for efficiency/stability of pairing and repair, especially *in vivo* when Rad51 filament must discriminate between many potential homologous templates. Perhaps this could suggest the reason why *in vivo* longer regions of homology are required for BIR to take place, as shown in ref. 24. Perhaps it is insignificant but the Authors could at least address the issue.

**[Authors’ Response #20]:** We thank the reviewer for having suggested this important point. Interestingly, our results showed that donor template D with 48 perfectly matched base pairs near the 3’ invading end of the DSB yielded an unexpectedly lower efficiency of BIR compared to donor template B with a cluster of mismatches starting at 1st bp near the 3’ end. Therefore, the regions containing mismatches every 6^th^ bp near the 3’ end must play a role in base-pairing and repair *in vivo*. The explanation of unexpected and interesting results on donor template B and D were part of the discussion.

**[Comment 21]: line 204** *However, none of the arrangements of 10 mismatches with every 6th base spacing was as efficient as a template with 10 mismatches, spaced every 10 base pairs, although in two cases the difference was not statistically significant.*

If the difference for two templates was not significant, then these two templates with clustered mismatches should be regarded as efficient as equally spaced control.

**[Authors’ Response #21]:** We agree with the reviewer and have added the revised the statement in the discussion.

**[Comment 22]: Iines 207-215** If the regions with many mismatches were not base-paired at all, we would expect to see BIR efficiencies comparable to the length of the remaining ~ 54 bases in perfectly-matched sub-regions; however, we have previously shown that a template that had 54 perfectly matched base pairs (i.e. equivalent to the segments of the templates with no mismatches) yielded a BIR rate less than 7% of a 108-bp region [24], whereas the efficiencies of the 6 templates we used were about 26% as successful (and where every 10th was 44%). So the regions containing mismatches every 6th nucleotide must play a role in base-pairing, but there is a significant reduction in efficiency. This penalty is less than the very strong barrier seen from in vitro strand exchange studies [10,30,31].

This part lacks clarity and it is difficult to follow the Author’s reasoning.

**[Authors’ Response #22]:** Given the additional findings about *in vitro* results, we have modified this discussion significantly.

**[Comment 23]: line 227** *...deleting Msh2 led to an overall reduction in repair efficiency for both the evenly- and unevenly- spaced templates so that any differences became statistically insignificant....*

Data shown in Fig. S2 demonstrate that differences between respective templates in *WT* and *msh2*Δ mutant backgrounds are not statistically significant so the Authors should not claim that deletion of *MSH2* caused reduction of repair. Why should it?

**[Authors’ Response #23]:** Although none of the pairwise comparisons between WT and *msh2*Δ for individual templates were statistically significantly different (Fig. S5), the differences between the WT and *msh2*Δ data as a whole were significant by a Mann-Whitney test (p = 0.004). We conclude that there is some other factor beyond the mismatch repair machinery that causes the unevenly-distributed mismatches to be less successful in BIR than the evenly-distributed controls.

**[Comment 24]:** .... *but a similar correlation with Tm persists.*
It is impractical to assess the correlation for a set of data that don’t differ significantly from each other (cf. Fig 5C).

**[Authors’ Response #24]:** Agreed, but we now note that by a Mann-Whitney test, msh2 and WT data as a whole are significantly different. We do not know the basis of this difference.

**[Comment 25]:** It would be informative to the reader if the Authors explained in more detail how their findings might be relevant to the role of Pol δ in meiosis.

**[Authors’ Response #25]:** Thank you for pointing this out. To focus more on current discussion on the role of Pol δ, we decided to delete this sentence in the discussion.

**Response to Reviewer #3**

**[Comments] Reviewer #3:** In the reviewed manuscript entitled “Repair of Mismatched Templates during Rad51-dependent Break-Induced Replication” the authors presented the results of their studies on the efficiency of break-induced replication (BIR), the homologous recombination subpathway. In their study they explored the influence of mismatches distribution in a donor template on the final repair product. By examining different templates containing the same number of mismatches but distributed unevenly, they were able to show the polar effect of insertion of the donor sequence into the final repair product. The mismatches located at the 3’ end of the invading strand were commonly replaced by the template sequence, but this tendency was gradually limited, and about 40 bp from the 3’ end, incorporation of the template sequence was rare. According to the authors, this effect was linked to the 3’-5’ exonucleolytic (proofreading) activity of polymerase δ, synthesizing DNA during repair; however, it had nothing to do with the proofreading activity per se, since 3’-5’ exonucleolytic activity was observed independently of mismatches presence or absence in the sequence. Authors call this activity “chewing back” the 3’ end of invading strand and claim that this step precedes the initiation of DNA synthesis using the donor template. Thus, the additional step during the initiation of BIR was recognized. The finding that donor strand undergoes resection is important; however, an indication of which enzyme is responsible for its processing is still open. The authors did not prove that, so they should moderate their statement and include alternative scenarios in the text or make a similar set of experiments as they described, using a set of strains with a polymerase variant impaired in proofreading activity. In the previous works, the researchers from the same laboratory showed that it is possibly not Exo1 activity; however, yeast Saccharomyces cerevisiae possesses broader resources of 3’-5’ exonucleases that should be considered. Interestingly, one of the findings is that the Msh2-dependent mismatch repair system seems not to contribute to BIR even when mismatches are present in the duplex created by the acceptor and donor strand. Presented data contribute to our understanding of the recombination process. However, the results of this study also underlined the differences between results obtained in the in vitro ad in vivo studies and showed that the model used to reveal the mechanism of recombination could influence the results by the length and sequence of the homologous region used in the attempt. The data obtained by the authors brought us closer to solving the complicated puzzle of the homologous recombination process, whose final effect is enzyme- and template-dependent.

**[Authors’ Response]:** We would like to express our sincere gratitude to Reviewer #3 for the constructive comments on our manuscript. As Reviewer #3 suggested, we created a set of strains with proofreading-defective DNA Polymerase δ mutant (*pol3-01*) for both evenly- and unevenly-mismatched templates. The results are added as a new Fig. 6 and added to the discussion of the new data in the manuscript.
