# Supplementary material for: Repair of mismatched templates during Rad51-dependent Break-Induced Replication
Source: PLoS Genet. 2022 Sep 2;18(9):e1010056. doi: 10.1371/journal.pgen.1010056 (PMC9477423; doi:10.1371/journal.pgen.1010056)
Supplement: Attachment — Submitted filename: Responses to Reviewers_JC_BIR_2022.docx [file pgen.1010056.s016.docx]

Responses to Reviewers

We were very pleased by the enthusiastic reception of our revised MS. We list below the disposition of the comments by Reviewer #2. We believe these changes should resolve any outstanding issues.

*Reviewer #2: Presented revised manuscript contains two sets of new results:
• analysis of pol3-01 mutant demonstrating that it is indeed the exonuclease activity of DNA polymerase delta that is responsible for extensive substrate degradation even when no mismatches are present
• in vitro D-loop assays with evenly and unevenly mismatched templates that complement presented data on the effect of mismatches on BIR in vivo
These are very valuable inclusions that greatly enhance the value of this manuscript.
However, some general conclusions are made based only on the subset of presented data and discussion of unexpected findings is very brief.*

*Comment 1. Generalized conclusions, understatements based on a subset of presented data. These can be very misleading to the reader.

line 23 These different arrangements of uneven mismatch distributions were in general less efficient in recombination than templates with evenly distributed mismatches.

2 (B, C) out of 6 were not less efficient, they were as efficient as evenly-spaced control.
The Authors acknowledge that

line 29 mismatch position-specific effects are also important

but continue to generalize without making reservations about B and C (also lines 235, 345, maybe others), and such imprecise statement will remain as take home message from this paper.*

[Response to comment 1a]: We have rephrased this statement (l. 26) to say: “Although 2 of the 6 arrangements we tested were nearly as efficient as the evenly-spaced reference, 4 were significantly less efficient.”

*line 25 A donor with all 10 mismatches clustered at the 3’ invading end of the DSB was not impaired compared to arrangements where mismatches were clustered at the 5’ end.

Understatement, donor B was not impaired not only when compared to donor D (which was one of 4 impaired as compared to the evenly-spaced control), but also when compared to the evenly-spaced control itself.*

[Response to comment 1b]: To be honest, we are confused by the reviewer. We are chided for not making reservations about B versus others (B not being statistically different from the evenly-spaced reference) and now are criticized for not distinguishing it from the control, although there is no statistically significant difference. We think the revised phrasing above is sufficient. The data are there for the reader to judge

*line 214 (also line 347) But unlike the results in vivo, there was no general reduction in product formation compared to the evenly-distributed controls in the in vitro assays.

Understatement, in fact BIR efficiency was increased for 4 out of 6 substrates, same for C and lower for E. No statistical significance shown for in vitro data (such as seen for in vivo BIR in Fig2C).*

[Response to comment 1c]: Statistical significance is now shown for part D of revised Figure 3.

We rephrased the sentence to say: “But unlike the results in vivo, there was no general reduction in product formation compared to the evenly-distributed controls in the in vitro assays. In fact 4 of the constructs showed increases.

*line 228 In general, the efficiency of BIR was lower in the absence of Msh2 and line 235 We conclude that there is some other factor beyond the mismatch repair machinery that causes the unevenly distributed mismatches to be less successful in BIR than the evenly-distributed controls

Both relate to the Authors previous response to Rev 1 comment 16.
I agree WT and delmsh2 sets may be different, but if so why the conclusion is that the difference is mediated by other factors than Msh2?

I am not convinced it is appropriate to describe data presented in Fig. S2 (as well as other data in this manuscript, as mentioned above) in terms of “data as a whole” or „in general”.
This obscures a fact that some donors with unevenly-spaced mismatches do appear to behave in a different, difficult to explain but, in my opinion, potentially very interesting way.*

[Response to comment 1d]: We disagree. We think it is important to discuss the outcomes as a group (i.e. none of the 6 arrangements were as efficient as the control) as well as individually (4 of the arrangements were statistically significantly different). We have tried not to obscure the differences among the 6 constructs.

*Comment 2.
line 282 ...cells lacking MLH1 or MSH2 still can extend and correct mismatches >30 bp from the 3’ invading end of the DSB...

Is the drop to 60% for incorporation of first mismatch for template A in delmsh2 meaningful?*

[Response to comment 2]: Given that there is no similar drop without Mlh1, we don’t think the drop for template A is likely to be significant.

*Comment 3.
line 293 Deleting Rad1 did not affect the BIR efficiency or mismatch incorporation of mismatches on both evenly- and unevenly-mismatched donor templates.

Generalisation based on one unevenly-mismatched donor. Why yRA275 and not yRA280 was used as evenly spaced control? Why E was chosen? It would be interesting to see if first mismatch is affected in donor A (as examined for MSH2) or B (as examined for MPH1).
Same representative subset of substrates should be examined for all mutants tested and their choice explained.*

[Response to comment 3]: We do not think it is a problem that we looked at Rad1 in an evenly spaced every 9^th^ versus every 10^th^ construct. Template E was chosen to look at a case where there were no mismatches in the first 1/3 of the sequence and to show here that Rad1-Rad10 were not the agents of the removal of sequence >25 nt from the 3’ end

*Comment 4.
line 293 This observation agrees with a previous study that Srs2 is required for bubble migration during BIR and deleting Srs2 promotes formation of toxic joint molecules from uncontrolled Rad51 binding to the intact donor, interfering with BIR completion.

Presented data do not relate to the mechanism of Srs2 activity. They show that in delsrs2 the frequency of BIR is too low to assess the effect of this helicase on BIR with mismatched templates.*

[Response to comment 4]: We agree. We have deleted the offending sentence beginning l. 308 and replaced it by “Consistent with previous observations, deleting Srs2 significantly reduced the repair efficiency (52) and prevented us from analyzing specific effects related to mismatch incorporation into the BIR product (Fig. S7).

*Comment 5.
line 322 These results support in vitro studies that have suggested that Rad51-mediated pairing does not have to begin at the 3’ end (9, 11, 12, 55, 56), but it is not clear why substrates with a well matched 3’ end should be less efficient.

Perhaps it is not that well matched 3' end is less efficient but that less matched further regions (that arise if mismatches are clustered away from 3'end) are important in vivo. This would be an important phenomenon that failed to be captured by in vitro assays.*

[Response to comment 5]: We think the statement as written should stand as is.

*Comment 6.
line 326 Previous in vitro studies had suggested that Rad51 was incapable of stably binding substrates in which there were fewer than 8 consecutive homologous base pairs....
and line 334 Here, we show that Rad51, aided by Rad54, can indeed create stable D-loops in vitro with 90 or 108 nt ssDNA substrates in which every 6th base is mismatched.....

Was Rad54 included in earlier cited in vitro studies? If not this would be another difference between previous and current work that should be discussed.*

[Response to comment 6]: We thank the reviewer for raising this point regarding Rad54. We have made changes in lines 373-377 to acknowledge the absence of Rad54 in earlier cited *in vitro* studies.

*Comment 7.
line 178 The differences among these templates cannot be attributed to a difference in thermal stability of base-pairing in the 108-bp region as measured by the calculated melting temperature (Tm) between complementary 108-nt DNA strands.... Interestingly, the slopes of the linear regression lines were nearly identical for the evenly- distributed controls .... as for the 6 unevenly spaced cases .... but the unevenly-distributed series lie below the controls.

Description of data presented in Fig.3 is very brief and it is not apparent how the Authors reach and what they mean (with respect to mismatched templates that are examined in this work) by the general conclusion presented in the Discussion :

line 341 Our data lend some support to the hypothesis that the success of strand pairing depends on the total number of base pairs that can be formed, or – more precisely – to the total energy of base pairing that is achieved.*

[Response to comment 7]: We thank the reviewer for the comment. We have expanded this part of the Discussion to say:

Our data lend some support to the hypothesis that the success of strand pairing depends on the total number of base pairs that can be formed, or – more precisely – to the total energy of base pairing that is achieved. Thus, among the set of 6 donors with different clustering of mismatches, those with a higher Tm tended to have higher BIR efficiencies. The relationship between template Tm is the same for the six unevenly-distributed donors compared to the evenly-distributed controls, but the set with clustered mismatches is repaired less efficiently. This downward shift is a feature of the *in vivo* process, as the *In vitro* analysis of these same templates shows a similar relationship between pairing efficiency and Tm but does not show a reduction of D-loop formation by unevenly-mismatched substrates relative to the evenly-matched controls (Fig. 3C).

*Comment 8.
line 341 It is also unclear what enforces the reduced success of these templates in vivo...

This is a very interesting finding that perhaps warrants further discussion. Other helicases, chromatin factors that could play a role and be examined in the future?*

[Response to comment 8]: We don’t think it is necessary to add more speculation.

*Comment 9.
Discussion of another very interesting result is also brief.
line 380 ... we entertain the idea that a small amount of residual 3’ to 5’ excision activity might remain...
On what basis?*

*line 383 ....there may be another activity that can accomplish this end-removal.
Such as what?*

[Response to comment 9]: We revised the sentence to say: Alternatively, there may be another exo- or endonuclease activity that can accomplish this end-removal.

*Comment 10.
line 132 ....but, unexpectedly, donor templates with majority of mismatches towards the 5’ end were statistically significantly lower than the evenly-distributed control.
Unclear, needs rephrasing.*

[Response to comment 10]: We now state: Unexpectedly, donor templates with majority of mismatches positioned towards the 5’ end were statistically significantly lower than the evenly-distributed control.
